# Multiple-locus variable-number tandem repeat analysis potentially reveals the existence of two groups of *Anaplasma phagocytophilum* circulating in cattle in France with different wild reservoirs

**DOI:** 10.1186/s13071-016-1888-4

**Published:** 2016-11-22

**Authors:** Thibaud Dugat, Gina Zanella, Luc Véran, Céline Lesage, Guillaume Girault, Benoît Durand, Anne-Claire Lagrée, Henri-Jean Boulouis, Nadia Haddad

**Affiliations:** 1 Agence nationale de sécurité sanitaire de l’alimentation, de l’environnement et du travail, Laboratoire de Santé Animale, UMR BIPAR, Université Paris-Est, Maisons-Alfort, France; 2 Agence nationale de sécurité sanitaire de l’alimentation, de l’environnement et du travail, Laboratoire de Santé Animale, Unité d’Epidémiologie, Université Paris-Est, Maisons-Alfort, France; 3 Fédération des chasseurs du Loiret, Orléans, France; 4 Agence nationale de sécurité sanitaire de l’alimentation, de l’environnement et du travail, Laboratoire de Santé Animale, Unité des Zoonoses Bactériennes, Université Paris-Est, Maisons-Alfort, France; 5 Ecole Nationale Vétérinaire d’Alfort, UMR BIPAR, Université Paris-Est, Maisons-Alfort, France

**Keywords:** *Anaplasma phagocytophilum*, France, Group A, Group B, MLVA, Reservoir, Red deer, Roe deer, VNTR, Wild boars

## Abstract

**Background:**

*Anaplasma phagocytophilum* is the causative agent of tick-borne fever, a disease with high economic impact for domestic ruminants in Europe. Epidemiological cycles of this species are complex, and involve different ecotypes circulating in various host species. To date, these epidemiological cycles are poorly understood, especially in Europe, as European reservoir hosts (i.e. vertebrate hosts enabling long-term maintenance of the bacterium in the ecosystem), of the bacterium have not yet been clearly identified. In this study, our objective was to explore the presence, the prevalence, and the genetic diversity of *A. phagocytophilum* in wild animals, in order to better understand their implications as reservoir hosts of this pathogen.

**Methods:**

The spleens of 101 wild animals were collected from central France and tested for the presence of *A. phagocytophilum* DNA by *msp2* qPCR. Positive samples were then typed by multi-locus variable-number tandem repeat (VNTR) analysis (MLVA), and compared to 179 previously typed *A. phagocytophilum* samples.

**Results:**

*Anaplasma phagocytophilum* DNA was detected in 82/101 (81.2%) animals including 48/49 red deer (98%), 20/21 roe deer (95.2%), 13/29 wild boars (44.8%), and 1/1 red fox. MLVA enabled the discrimination of two *A. phagocytophilum* groups: group A contained the majority of *A. phagocytophilum* from red deer and two thirds of those from cattle, while group B included a human strain and variants from diverse animal species, i.e. sheep, dogs, a horse, the majority of variants from roe deer, and the remaining variants from cattle and red deer.

**Conclusions:**

Our results suggest that red deer and roe deer are promising *A. phagocytophilum* reservoir host candidates. Moreover, we also showed that *A. phagocytophilum* potentially circulates in at least two epidemiological cycles in French cattle. The first cycle may involve red deer as reservoir hosts and cattle as accidental hosts for Group A strains, whereas the second cycle could involve roe deer as reservoir hosts and at least domestic ruminants, dogs, horses, and humans as accidental hosts for Group B strains.

**Electronic supplementary material:**

The online version of this article (doi:10.1186/s13071-016-1888-4) contains supplementary material, which is available to authorized users.

## Background

*Anaplasma phagocytophilum* is a zoonotic intragranulocytic alpha-proteobacterium transmitted by ticks belonging to the genus *Ixodes*: *I. ricinus* in Europe, *I. scapularis* in Eastern USA, *I. pacificus* and *I. spinipalpis* in Western USA, and *I. persulcatus* in Asia and Russia [1]. It infects a large range of hosts worldwide, including humans, wild and domestic ruminants, horses, domestic carnivores, birds and rodents [1].

*Anaplasma phagocytophilum* is the causative agent of granulocytic anaplasmosis in humans, horses, dogs, and occasionally cats, and tick-borne fever (TBF) in domestic ruminants. The epidemiology of *A. phagocytophilum* infection differs greatly between the USA and Europe. In the USA, human granulocytic anaplasmosis (HGA) is an increasing public health problem (the CDC reported 2,389 human cases in 2012 [2]), whereas no TBF cases have been described to date in this country. Conversely, HGA appears to be rarer in Europe (even though the number of reported cases has increased during recent years, probably linked in part to improved surveillance [3, 4]), whereas a high number of TBF cases have been described in both cattle and sheep, causing significant economic losses [1].

Epidemiological cycles of *A. phagocytophilum* are complex and involve different ecotypes, vectors, and mammalian host species. To date, these epidemiological cycles are not completely understood, especially in Europe, as European reservoir hosts of the bacterium have not yet been identified. Red deer (*Cervus elaphus*) and roe deer (*Capreolus capreolus*) have been suspected to be potential *A. phagocytophilum* reservoir hosts [1]. However, recent studies strongly suggest that roe deer are not reservoir hosts for human, dog, horse, or domestic ruminant variants [5–8]. For this reason, we and other authors have hypothesized that roe deer could be reservoir hosts for their own *A. phagocytophilum* variants [1]. Additionally, other data indicate that red deer could be reservoir hosts for domestic ruminant variants, but not for human, dog, or horse variants [6–9]. Wild boars (*Sus scrofa*) are also suspected to be reservoir hosts for human *A. phagocytophilum* variants [8, 10]. Finally, several rodent species have been suspected as reservoir hosts for *A. phagocytophilum*, but - at least at the scale of published studies - they appear to be involved in an epidemiological cycle independent from those involving ruminants, in which rodents are the only mammalian hosts [11, 12].

The role of wild animals in *A. phagocytophilum* epidemiological cycles must be clarified in order to facilitate the development of relevant prevention and control measures. In a previous study, we developed a multiple-locus variable-number tandem repeat (VNTR) analysis (MLVA) technique in order to investigate *A. phagocytophilum* epidemiology and genetic diversity [6]. In the present study, our objective was to investigate the presence, the prevalence, and the genetic diversity of *A. phagocytophilum* obtained from wild animals, in order to better understand whether they can be implicated as reservoir hosts of this pathogen. To address our objective, the presence of *A. phagocytophilum* DNA was determined in wildlife from central France by real-time PCR, and genetic diversity was explored using MLVA. Resultant sample diversity was then analyzed and compared to current *A. phagocytophilum* diversity data.

## Methods

### Animal sampling

Spleens from 49 red deer (*Cervus elaphus*), 29 wild boars (*Sus scrofa*), 21 roe deer (*Capreolus capreolus*), 1 red fox (*Vulpes vulpes*), and 1 river rat (*Myocastor coypus*) were collected between 2009 and 2015 from 21 different areas around central France (Additional file 1: Table S1). Spleens were collected from gunshot animals, and stored at -80 °C before analysis.

### DNA extraction

For DNA extraction, the NucleoSpin® Tissue kit (Macherey-Nagel, Bethlehem, USA) was used according to the manufacturer’s instructions. DNA extracts were then stored at -20 °C prior to testing.

### Detection of *A. phagocytophilum* DNA by *msp2* qPCR

*Anaplasma phagocytophilum* DNA was detected by qPCR, targeting a 77 bp fragment of the *msp2* (*major surface protein 2*) gene as previously described by Courtney et al. [13]. Water (molecular biology grade) was used as a negative control. DNA extracted from the Human Webster strain was used as a positive control [14]. qPCR reactions were performed in triplicate, and the mean value was used in the following analyses.

### MLVA

The MLVA protocol was conducted as previously described by Dugat et al*.* [6]. Obtained MLVA profiles were compiled in a database already containing the profiles of 179 *A. phagocytophilum* samples from different animal hosts typed in previous studies: cattle (125, of which 25 had aborted), sheep (7), roe deer (15), red deer (4), reindeer (1), horses (2), dog (1), *Rhipicephalus* spp. (25), and the Human Webster strain [6, 15].

### Statistical analysis

Confidence intervals (95% CI) were estimated using an exact binomial distribution. The association between qPCR results and species was studied in red deer, roe deer and wild boars. The association between PCR results and sex and age class (juveniles: ≤ 1 year, adults: > 1 year) was tested by species. All statistical analyses were performed by using R software [16].

### Clustering analysis

MLVA clustering was performed using the BioNumerics software package version 7.5 (Applied-Maths, Sint-Martens-Latem, Belgium). Data were analyzed as a character dataset and the similarity matrix was computed using a categorical distance. Based on this similarity matrix, the Minimum Spanning Tree (MST) graphing algorithm was used to represent the relationships between strains. The priority rule for constructing MSTs was set so that the type which had the highest number of single-locus variants would be linked first. A cut-off value of maximum differences of one VNTR was applied to define clonal complexes under the MST method.

## Results

### Detection of *A. phagocytophilum* DNA by *msp2* qPCR

In total, 82 of 101 animals (81.2%; 95% CI: 72.2–82.3%) were *msp2* qPCR-positive: 48/49 red deer (98%; 95% CI: 89.9–99.1%), 20/21 roe deer (95.2%; CI: 76.2–99.9%), 13/29 wild boars (44.8%; 95% CI: 26.4–64.3%), 1/1 red fox, while the single river rat tested provided a negative result (Table 1, Additional file 1: Table S1).

**Table 1:** *Anaplasma phagocytophilum* infection prevalence in each animal species

Animal species	No. of *msp2-*positive/Total no. examined	Positive (%)	95% CI
Red deer (*Cervus elaphus*)	48/49	95.2	89.2–100
Roe deer (*Capreolus capreolus*)	20/21	92.5	81.2–100
Wild boar (*Sus scrofa*)	13/29	44.8	26.7–62.9
Red fox (*Vulpes vulpes*)	1/1	nd	nd
River rats (*Myocastor coypus*)	0/1	nd	nd
Total	82/101	81.2	73.6–88.8

*Abbreviation*: *CI* confidence interval; *nd* not determined

### Statistical analysis

Three variables (i.e. sex, age and animal species) were tested in order to determine whether they were associated with a higher probability of animal infection. The red fox and river rat samples were excluded from the statistical analysis as there was only one sample from each species. A significant association was found between the host species and positive *A. phagocytophilum* qPCR results. Red deer and roe deer were significantly more frequently infected than wild boars (Fisher’s exact test, *χ* ^2^ = 27.136, *P* < 0.001). Finally, no association was found between sex and age and *A. phagocytophilum* infection.

### MLVA analysis

**Fig. 1:**
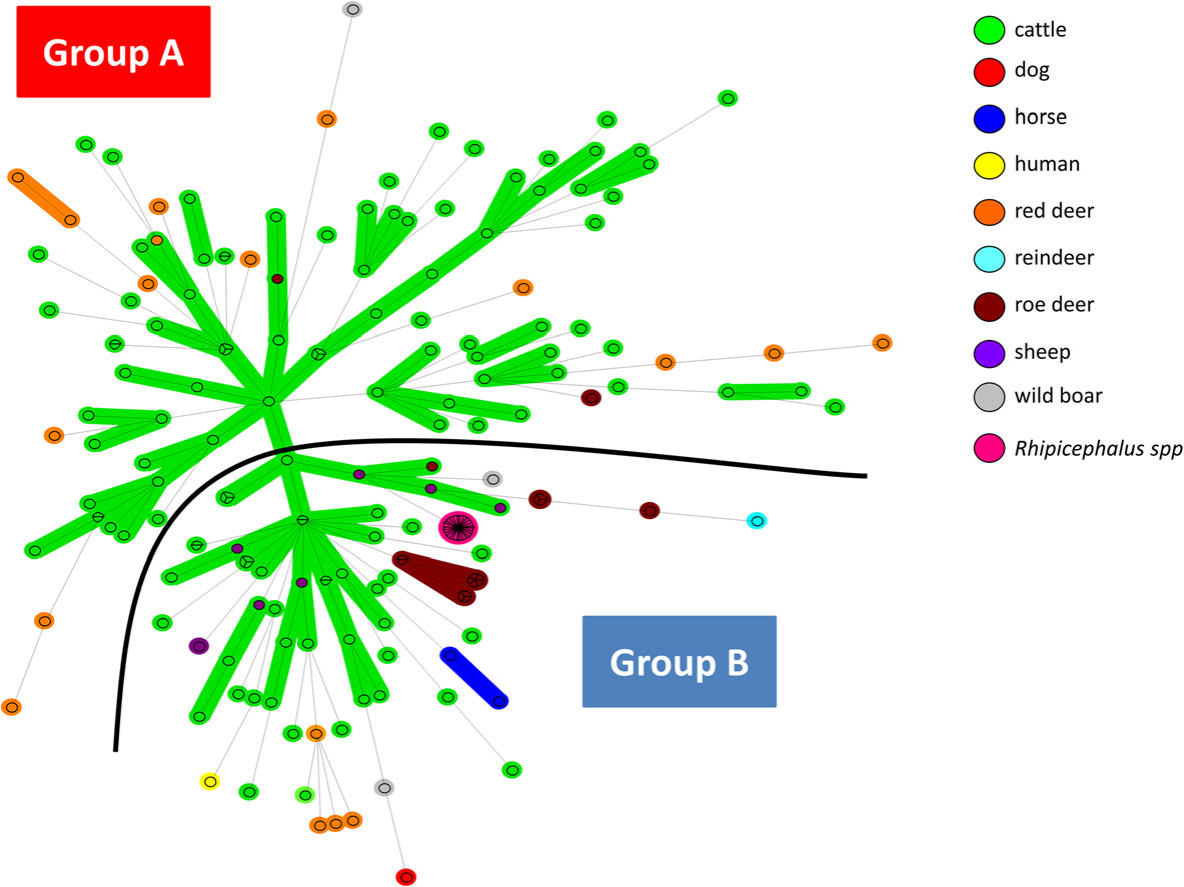
Minimum spanning tree of the 198 *A. phagocytophilum* database samples according to their host species. Each circle represents a unique MLVA profile. The number of circle partitions corresponds to the number of *A. phagocytophilum* samples with the same genotype. Circles connected by a shaded background and tick lines differ by a maximum of one of the five VNTR markers, and could be considered as a “clonal complex”. The length of each branch is proportional to the number of differences. Each animal host species is represented by a specific color in the circle

We obtained complete MLVA profiles for 19/82 positive samples (typability: 23.2%), including 14 from red deer, two from roe deer and three from wild boars (Additional file 2: Table S2). These 19 profiles have never before been published [6, 15]. The cut-off value of samples generating complete profiles varied from 21.6 to 37.4 (Additional file 2: Table S2). Profiles were compiled and added to a database which already contained 179 MLVA profiles from previous studies [6, 15]. The 198 resulting profiles (available in Additional file 3: Table S3) were then represented on an MST (Fig. 1).

**Fig. 2:**
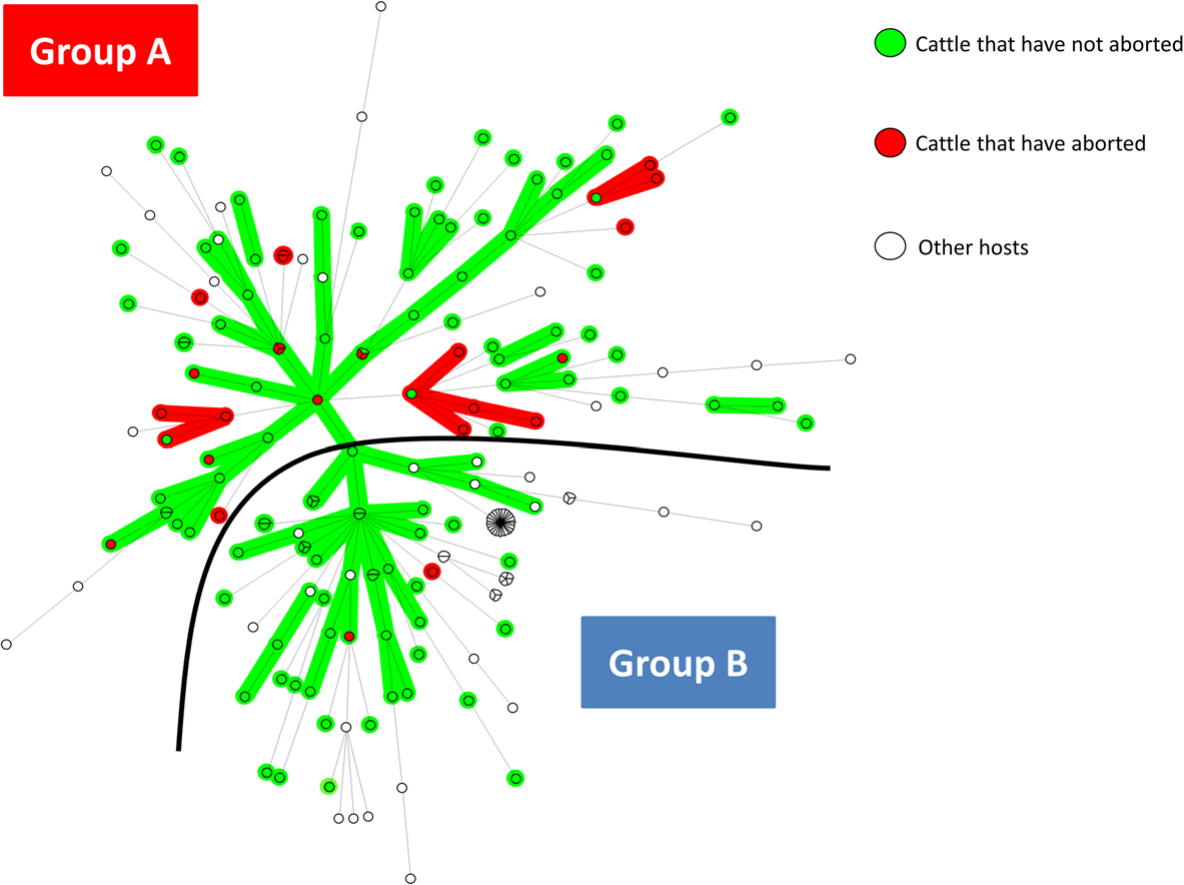
Minimum spanning tree of the 198 *A. phagocytophilum* samples according to the abortion status of their hosts. Each circle represents a unique MLVA profile. The number of circle partitions corresponds to the number of *A. phagocytophilum* samples with the same genotype. Circles connected by a shaded background and tick lines differ by a maximum of one of the five VNTR markers, and could be considered as a “clonal complex”. The length of each branch is proportional to the number of differences. *Anaplasma phagocytophilum* obtained from cattle that have aborted are red, and those from cattle that have not are green. *Anaplasma phagocytophilum* obtained from other host species are represented in white

The MST could be divided into two groups: group A and group B. Group A included the majority of *A. phagocytophilum* from cattle (83/123), and red deer (14/18), and only four other variants: 1/3 from wild boar, and 2/17 from roe deer. Group A also contained the majority of variants obtained from cattle having aborted, 23/25 (92%) (Fig. 2). Group B included all samples from *Rhipicephalus* spp. (25/25), sheep (7/7), horses (2/2), the dog (1/1), the reindeer (1/1), the majority of *A. phagocytophilum* from roe deer (15/17) and wild boar (2/3), approximately one third of cattle samples (40/123), and the remaining samples from red deer (4/18), and the human strain HZ (1/1) (Table 2, Fig. 1). *Anaplasma phagocytophilum* from red deer and roe deer tended to lie towards the periphery of the MST (Fig. 1). Finally, variants did not seem to cluster according to their geographical location.

**Table 2:** Number of *A. phagocytophilum* samples belonging to group A or B

Host species	No. of samples in group A (%)	No. of samples in group B (%)	Total no. of samples
Cattle	83 (67.0)	40 (33.0)	123
Sheep	0 (0)	7 (100)	7
Red deer	14 (77.8)	4 (22.2)	18
Roe deer	2 (11.8)	15 (88.2)	17
Reindeer	0 (0)	1 (100)	1
Wild boar	1 (33.0)	2 (67.0)	3
Dog	0 (0)	1 (100)	1
Horse	0 (0)	2 (100)	2
Human	0 (0)	1 (100)	1
*Rhipicephalus* spp.	0 (0)	25 (100)	25
Total	100 (51.0)	98 (49.0)	198

## Discussion

To date, *A. phagocytophilum* reservoir hosts have not yet been clearly identified in Europe. Several wild animal species, particularly red and roe deer, have been suspected to play this role. In particular, several studies suggested that red deer are reservoir hosts for *A. phagocytophilum* transmission to sheep [17, 18]. In this work, our objective was to study the presence and the genetic diversity of *A. phagocytophilum* in wild animals, in order to explore the role of these animals in *A. phagocytophilum* epidemiological cycles.

In the first part of the study we assessed the presence of *A. phagocytophilum* in different wild animals. We report here for the first time the presence of *A. phagocytophilum* DNA in a red fox in France [19]. The role of this animal species in *A. phagocytophilum* epidemiological cycles has been poorly studied to date. Unfortunately, only one red fox sample was available, and no MLVA profile could be obtained due to poor DNA quality. More samples are required in order to investigate the role of this animal in *A. phagocytophilum* epidemiological cycles and to determine whether this positive result was related to chance or to real high infection levels in red foxes.

Very high *A. phagocytophilum* DNA prevalence rates were observed in red (98%) and roe deer (95.2%). These results are consistent with previous studies, where *A. phagocytophilum* DNA has been detected in up to 87.5 and 98.9% of red and roe deer, respectively [19]. Moreover, our study was the first detection of *A. phagocytophilum* DNA in wild boars from France. *Anaplasma phagocytophilum* DNA was detected in 13/29 wild boars (44.8%). This infection rate was much higher than expected, as it varied from 0.97 to 12% in wild boars from previous European studies [19, 20]. This result could be explained by high infection pressure, as we observed very high prevalence of *A. phagocytophilum* infection in red deer and roe deer in this area. This hypothesis is consistent with results from two other investigations studying *A. phagocytophilum* infection in wild boars and wild deer when occupying the same geographical habitat. In the first study, carried out in Spain, only 6/20 (30%) red deer were PCR-positive for *A. phagocytophilum*, and none of the 18 wild boars tested were positive [21]. In the second study, performed in Japan, 5/32 (15.6%) sika deer (*Cervus nippon*) were positive and only 2/56 (3.6%) of wild boars tested were PCR-positive for *A. phagocytophilum* [22]. In this study, the ratio of wild boar infected/deer infected was 1/5, compared to approximately 1/2 in our study, suggesting that a high infection pressure leads to higher wild boar infection rates. Taken together, these results indicate that red deer and roe deer are better candidates as *A. phagocytophilum* reservoir hosts, compared to wild boars. This is consistent with the work of Galindo et al*.* [23] who have questioned the role of wild boars as *A. phagocytophilum* reservoir hosts by demonstrating that their immune system quickly eliminates *A. phagocytophilum* infection.

In the second part of this study, we used an MLVA approach to explore in detail which species might act as reservoir hosts for cattle infecting strains. We obtained complete MLVA profiles for only 19/81 *msp2* qPCR-positive samples. This low number could not be explained by too little (or too much) DNA, as the samples which generated complete profiles had ct values varying from 21.6 to 37.4. The most likely hypothesis is that this result was linked to poor quality DNA, probably due to non-ideal sampling conditions following hunting expeditions, as animals may have been dead for many hours before sampling, and/or the samples may have been conserved at ambient temperature for several hours prior to freezing.

Using MLVA, we identified two *A. phagocytophilum* variant groups. Variants clustered according to their host species, and not according to their geographical location, which therefore excluded any associations between this variable and profile distribution. Indeed, Group A contained the majority of *A. phagocytophilum* from red deer and cattle, and only four samples from other animals. Group B comprised variants from many more species, including variants from the dog, two horses, the single human strain, all sheep, the vast majority of variants from roe deer and wild boars, and a few variants from cattle and red deer. Cattle seemed to be less frequently infected by *A. phagocytophilum* belonging to group B but this observation requires confirmation. Our results are in agreement with other studies which have already reported clustering of *A. phagocytophilum* from red deer and cattle into one group (cluster 1), and of *A. phagocytophilum* from roe deer into another group (cluster 2) [8, 24], thus confirming the existence of these two clusters in France.

Finally, *A. phagocytophilum* variants from red deer and roe deer were located at the periphery of the MST, which could be explained by at least two cumulative elements. First, contact occurs more frequently between cattle than between cattle and wild ruminants, favoring exchanges between cattle over those between cattle and wild ruminants. Secondly, a sampling bias in our study resulted in approximately three times more bovine samples than deer samples. This bias could have led to an underestimation of *A. phagocytophilum* exchanges between domestic and wild ruminants, and between wild ruminants, thus *A. phagocytophilum* from these species could perhaps be typed in a more marginal position. Precisely positioning *A. phagocytophilum* from wild ruminants will require larger cohorts.

Our results suggest the existence of at least two *A. phagocytophilum* epidemiological cycles in French cattle. The first cycle may involve red deer as reservoir hosts and cattle as major accidental hosts for group A strains. In addition, the vast majority of variants obtained from cattle having aborted (92%) belonged to group A, suggesting that red deer are the principal reservoir hosts of *A. phagocytophilum* involved in cattle abortions, and that the strains belonging to this group could be more harmful to cattle than strains belonging to group B, which seem more ubiquitous in their host tropism. The second epidemiological cycle could involve roe deer as reservoir hosts and at least domestic ruminants, dogs, horses, and humans as accidental hosts for group B strains. However, as group B strains seemed to infect a larger range of hosts, we cannot exclude that (an)other animal species could be involved as reservoir hosts for these strains. In particular, the role of sheep needs to be investigated, as this species has previously been thought to represent a reservoir host for *A. phagocytophilum* [1, 25]. Moreover, even if rodents seem to be involved in independent epidemiological cycles in some European countries [1], their role in group A and group B strain epidemiology in France remains to be clarified.

A low proportion of red deer (22.8%) and roe deer (11.8%) were infected by group B and group A variants respectively. At first glance, this could be considered as contradictory with regard to the hypothesis that red deer could be reservoirs hosts for group A variants, and roe deer for group B variants. But as both species share the same ecosystem, it is highly conceivable that both of them could be accidentally infected by variants infecting preferentially, but not exclusively, the other species of wild ruminants. In this context, wild boars, which are omnivorous animals and scavenge the carcasses of both species, could represent accidental hosts for both variants.

In order to confirm this hypothesis, more wild and domestic animal samples originating from different regions in France should be tested. This would also confirm whether group B variants have a higher propensity to infect more species, and conversely, whether group A variants are more adapted to red deer and cattle. In addition, these wild ruminants could suffer from *A. phagocytophilum* infection at either individual and/or population levels, which may then impact their role in *A. phagocytophilum*’s epidemiological cycle. This hypothesis has never been investigated, and thus merits further attention, especially in the context of multiple infections.

## Conclusions

This study is the first report of *A. phagocytophilum* DNA in a red fox and in wild boars in France. We also report a very high prevalence of *A. phagocytophilum* infection in red deer and roe deer. Moreover these animals present a high bacterial load. These results strongly suggest that red deer and roe deer are reservoir hosts of *A. phagocytophilum*. The MLVA approach enabled the description of two *A. phagocytophilum* variant groups. Group A strains could circulate within red deer as reservoir hosts and cattle as accidental hosts, whereas Group B strains could circulate within roe deer (and/or other animal species) as reservoir hosts and at least domestic ruminants, dogs, horses, and humans as accidental hosts. Confirming this hypothesis will aid the development of relevant control measures for domestic ruminant *A. phagocytophilum* strains.

## Additional files

Additional file 1: Table S1. Characteristics and qPCR results for the 101 *A. phagocytophilum* wildlife samples used in this study. (XLSX 17 kb)
Additional file 2: Table S2. MLVA profiles of the 19 *A. phagocytophilum* wildlife samples typed in this study. (XLSX 13 kb)
Additional file 3: Table S3. MLVA profiles of the 198 *A. phagocytophilum* samples included in this study. (XLSX 22 kb)

## Abbreviations

CDC: Centers for disease control and prevention
HGA: Human granulocytic anaplasmosis
MLVA: Multiple-locus variable-number tandem repeat analysis
MST: Minimum spanning tree
TBF: Tick-borne fever
UPGMA: Unweighted pair group clustering method with arithmetic mean
VNTR: Variable number tandem repeat
